# Relationship of maximum walking speed with peak oxygen uptake and anaerobic threshold in male patients with heart failure

**DOI:** 10.1007/s00380-023-02289-y

**Published:** 2023-07-26

**Authors:** Masahiro Koen, Yoshiaki Kubota, Miwa Tokita, Kazuyo Kato, Hiroshi Takahashi, Koichi Akutsu, Kuniya Asai, Hitoshi Takano

**Affiliations:** Department of Cardiovascular Medicine, Nippon Medical School, 1-1-5, Sendagi, Bunkyo-ku, Tokyo, 113-8603 Japan

**Keywords:** Cardiopulmonary exercise testing, Peak oxygen uptake, Anaerobic threshold, Cardiac rehabilitation, Heart failure

## Abstract

This retrospective observational study aimed to examine the relationships of maximum walking speed (MWS) with peak oxygen uptake (peak VO_2_) and anaerobic threshold (AT) obtained by cardiopulmonary exercise testing (CPX) in patients with heart failure. The study participants were 104 consecutive men aged ≥ 20 years who had been hospitalized or had undergone outpatient care at our hospital for heart failure between February 2019 and January 2023. MWS was measured in a 5-m section with a 1-m run-up before and after the course. Multivariable analysis was used to examine the association between MWS and peak VO_2_ and AT by CPX. The Pearson correlation coefficient showed that MWS was positively correlated with percent-predicted peak VO_2_ and percent-predicted AT (r = 0.463, *p* < 0.001; and r = 0.485, *p* < 0.001, respectively). In the multiple linear regression analysis employing percent-predicted peak VO_2_ and percent-predicted AT as the objective variables, only MWS demonstrated a significant positive correlation (standardized β: 0.471, *p* < 0.001 and 0.362, *p* < 0.001, respectively). Multiple logistic regression analyses, using an 80% cutoff in percent-predicted peak VO2 and AT, revealed that only MWS was identified as a significant factor in both cases (odds ratio [OR]: 1.239, 95% confidence interval [CI]: 1.071–1.432, *p* = 0.004 and OR: 1.469, 95% CI: 1.194–1.807, *p* < 0.001, respectively). MWS was correlated with peak VO_2_ and AT in male patients with heart failure. The MWS measurement as a screening test for exercise tolerance may provide a simple means of estimating peak VO_2_ and AT in heart failure patients.

**Supplementary Information:**

The online version contains supplementary material available at 10.1007/s00380-023-02289-y.

## Introduction

Cardiopulmonary exercise testing (CPX) is a highly objective and reproducible form of testing for assessing exercise tolerance in patients with myocardial infarction and heart failure [1, 2]. Peak oxygen uptake (peak VO_2_), anaerobic threshold (AT), VO_2_/load, and minute ventilation/carbon dioxide production slope (VE/VCO_2_ slope), all of which are obtained from CPX, are closely related to worse outcomes and poor prognosis in patients with heart failure [3–6]. Moreover, AT is defined by the exercise level at which VE begins to increase exponentially relative to the increase in VO_2_ and is thought to reflect the limit of aerobic metabolism [7]. AT is important in safe and efficient cardiac rehabilitation and is used to determine loads in cardiac rehabilitation [8].

On the other hand, previous studies have shown that gait speed positively correlates with survival [9, 10]. Gait speed can be measured easily and quickly in any setting, requiring only a stopwatch without the need for specialized techniques, equipment, or expert knowledge. Methods for measuring gait speed include the 5-m walk test, 6-min walk test (6MWT) [11, 12], and shuttle walking test [13, 14]. The 5-m walk test, which typically uses comfortable gait speed, has been adopted as a criterion for assessing frailty and sarcopenia [15, 16]. Frailty and sarcopenia are, in turn, used as prognosticators for older adults [15, 17], indicators of acute coronary syndrome [18], and prognosticators of heart failure [19].

Furthermore, maximum walking speed (MWS), which is correlated with muscle strength, is an effective predictor of motor function in older adults [20]. MWS has been shown to be a potentially useful prognosticator of cardiovascular disease; older adults with a slow MWS present a high incidence of cardiovascular death [21].

Although MWS is not a common measurement method, and there are few reports on its use in patients with heart failure, MWS is a potential indicator of prognosis and exercise tolerance in these patients. To the best of our knowledge, no study has directly demonstrated the association of MWS with peak VO_2_ or AT in patients with heart failure. Therefore, we aimed to examine the relationships between 5-m gait speed and indicators obtained from CPX in patients with heart failure.

## Participants/materials and methods

### Participants

This retrospective observational registry study involves 104 men aged ≥ 20 years who had been hospitalized or had undergone outpatient care at our hospital for cardiovascular diseases and had been diagnosed with heart failure between February 2019 and January 2023. The participants had received optimal pharmacotherapy and could reliably visit our hospital for outpatient care by walking independently without using prostheses (such as canes) or wheelchairs. The diagnosis of heart failure was based on the Framingham criteria [22]. We proposed CPX to patients for evaluation of exercise tolerance and performed it on those who agreed. Consent regarding the use of data for research purposes was obtained in writing during CPX or cardiac rehabilitation. Additionally, the Institutional Review Board of our hospital approved the present study, and consent was obtained via an opt-out method. The study was conducted according to the Declaration of Helsinki guidelines.

The reason for focusing on males is that this study also measured grip strength, and grip strength varies by sex. From this fact, we concluded that MWS is also likely to vary by sex; thus, we excluded women.

### Collection of data

Data were collected via inspection of medical records and diagnostic interviews from instances where CPX was performed. We collected data on the following from clinical assessments and tests in daily clinical practice: age, date of heart failure onset, age at diagnosis, the reason for diagnosis, symptoms at enrolment, medical history (hypertension, diabetes, dyslipidemia, and cardiovascular events), exercise habits, test findings (coronary angiography findings, electrocardiography [ECG], paroxysmal atrial fibrillation, echocardiography, and blood test data), pharmacotherapy content, 5-m gait speed, and hand grip strength.

Hypertension was defined as systolic blood pressure ≥ 140 mmHg, diastolic blood pressure ≥ 90 mmHg, or current use of antihypertensive drugs. Dyslipidemia was defined as a low-density lipoprotein cholesterol level of ≥ 140 mg/dl, a triglyceride level of ≥ 150 mg/dl, or current use of antihyperlipidemic drugs. Diabetes was defined as an early-morning fasting glucose level of ≥ 126 mg/dl, a glucose level of ≥ 200 mg/dl at 2 h after a 75-g oral glucose tolerance test, or a blood glucose level of ≥ 200 mg/dl, regardless of measurement timing, and a hemoglobin A1c level of ≥ 6.5% or current use of antidiabetic drugs. Atrial fibrillation and atrial tachycardia were defined as paroxysms or continuity recorded in ECG. The left ventricular ejection fraction was calculated with the Teichholz formula or the modified Simpson method in echocardiography. For clinical data, we used the data from the most recent CPX in which the patient’s hemodynamics were stable. Smoking status was classified as “yes” if the patient had been smoking at the time of the study or had smoked previously. Other diseases were examined through the inspection of medical records and diagnostic interviews. Body mass index was calculated based on height and weight at the time of testing. Hand grip strength was measured twice on each side, and the maximum value was used.

### CPX

After the patients provided consent, we performed CPX on an outpatient basis to determine the appropriate load for exercise tolerance assessment and cardiac rehabilitation. For patients previously hospitalized, CPX was performed 30 days after discharge. An exercise test was performed on a cycle ergometer (Strength Ergo 8; Mitsubishi Electric Engineering Co., Ltd., Tokyo, Japan) coupled with a cardiopulmonary gas exchange system (Aero Monitor AE-310S; Minato Medical Science Co., Japan).

CPX was performed according to the following protocol. The device was recalibrated with a calibration gas composed of gases of known concentrations prior to each trial. Participants warmed up at 0–20 watts, with the wattage increased at 10–20-W intervals every minute thereafter by a ramp method. An exercise stress test electrocardiograph (STS-2100, Nihon Kohden Corporation, Japan) was utilized to continuously record heart rate and rhythm during the exercise stress test. Respiratory gas exchange was measured by the breath-by-breath method to identify peak VO_2_. AT was calculated with the V-slope method. The angular velocity during the test was aimed at 50–60 rpm, and a minimum of 50 rpm was maintained. Borg scale scores (6 [no exertion at all]–17 [maximal exertion]) were obtained after the exercise. Based on reports that a percent-predicted peak VO_2_ of ≥ 80% is equivalent to New York Heart Association class I [23], and percent-predicted peak VO_2_ or AT < 80% was defined as impaired exercise tolerance.

Exclusion criteria for this study were established based on the criteria of usual exercise stress tests such as CPX and treadmill stress testing. In accordance with the Japanese Circulation Society’s “2018 Guideline on Diagnosis of Chronic Coronary Heart Diseases [24],” we established the following exclusion criteria: acute phase of myocardial infarction at the time of exercise testing; high-risk unstable angina; poorly controlled arrhythmia; symptomatic severe aortic stenosis; acute or severe heart failure; acute pulmonary embolism or pulmonary infarction; acute myocarditis or pericarditis; consent being difficult or impossible to obtain due to serious cardiovascular lesions such as aortic dissection combined with cerebrovascular disease, dementia, or mental illness; or participation being deemed unsuitable by the attending physician, the principal investigator, or a sub-investigator. Additionally, participants with disorders strongly associated with reduced gait speed in CPX (dementia, neurological diseases, orthopedic disorders involving movement disorder, and other disorders impairing gait due to other forms of pain) were excluded from the analysis.

### Measurement of MWS

MWS was measured immediately before CPX; to do so, we referred to the sarcopenia/frailty testing and methods of a previous study [21]. MWS was measured in 5-m sections by two independent physicians as observers. A 1-m run-up was established before and after the course. To account for potential differences in walking proficiency among participants, the examiner thoroughly explained to the participants the style of gait to be used for the trials, showed them an example, and had them engage in one practice walk prior to walking speed measurement. To measure time, the examiner walked side-by-side with the participant, started a digital stopwatch (HS-3C-8AJH; Casio Computer Co. Ltd., Tokyo, Japan) the moment that any part of the participant’s body crossed the starting line, and stopped the stopwatch the moment the participant crossed the finish line (for all participants, measurement started and stopped the moment their toes crossed the line). Participants were instructed to always have one foot on the ground. To make measurements more accurate, the examiner measured the participant’s gait speed twice and used the mean of these two measurements as the test value.

### Statistical analyses

For patient background factors, continuous variables were expressed as mean ± standard deviation for normally distributed data or median [25th, 75th percentiles] for non-normally distributed data. Two variables were expressed as the number of subjects and proportion. Continuous variables were performed using Welch’s t-test for normally distributed variables and the Mann–Whitney U test for non-normally distributed variables. Comparisons between two variables were performed with the chi-square test. Following confirmation of a distribution with the Kolmogorov–Smirnov test, correlations between continuous variables were assessed with the Pearson correlation coefficient. Additionally, multiple linear regression and multiple logistic regression analyses were performed using explanatory variables that were correlated or tended to be correlated in simple regression analysis with percent-predicted peak VO2 and percent-predicted AT as objective variables.

In addition, we added an examination using propensity score matching (PSM) between the high and low MWS groups to minimize the influence of confounding variables that may lead to biased results. We selected the variables to generate propensity scores based on the results of the pre-matching two-group comparison. With a caliper width of 0.2 × standard deviation and a one-to-one closest neighbor matching algorithm, the propensity score was calculated using a multivariate logistic regression model. The degree of PSM was measured using a standardized mean difference. Furthermore, the relationship of MWS with peak VO_2_ and AT in this matched pair was examined using the Pearson correlation coefficient and logistic regression analysis.

All the statistical analyses were performed with Statflex ver. 7 (Artec Co., Ltd., Osaka, Japan). All *p* values were two-tailed, with *p* < 0.05 considered significant.

## Results

### Baseline characteristics

**Fig. 1:**
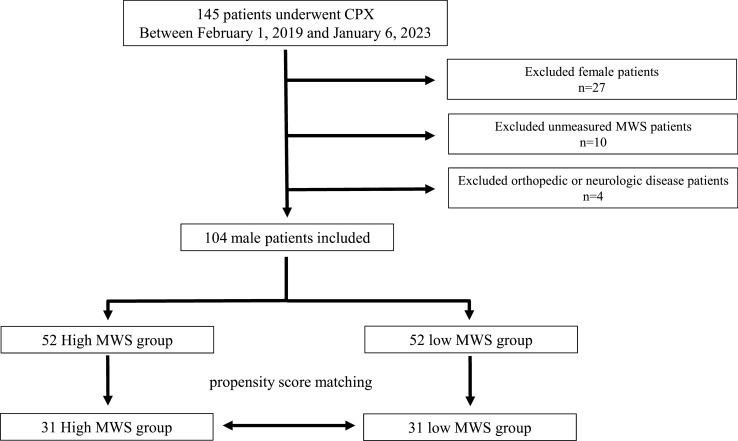
Flow chart of the screening and enrollment of study participants. CPX, cardiopulmonary exercise testing; MWS, maximum walking speed

Between February 1, 2019 and January 6, 2023, 145 patients underwent CPX for exercise tolerance assessment at our hospital. Twenty-seven patients were excluded because they were female. An additional 10 patients had an unmeasured MWS, and 4 patients were excluded because of gait difficulty due to orthopedic or neurologic disease. Finally, 104 male patients were enrolled. The flow chart is reported in Fig. 1.

Table 1 shows the baseline characteristics for the entire study population. The mean age of the participants was 60.2 ± 13.6 years. The mean left ventricular ejection fraction was 53.5 ± 14.2%. Ischemic heart disease was present in 71.2% of participants, while sinus rhythm was present in 96.2%. Medications being taken by the participants included calcium blockers (21.2%), beta-adrenergic blocking agents (84.6%), angiotensin-converting enzyme inhibitors or angiotensin receptor blockers (76.0%), and loop diuretics (11.5%).

**Table 1:** Clinical characteristics of the study population

Characteristics	All patients (n = 104)	High MWS (n = 52)	Low MWS (n = 52)	*p* value
Age, years	60.2 ± 13.6	54.8 ± 12.6	65.6 ± 12.5	< 0.001
Height, m	1.68 ± 0.07	1.70 ± 0.06	1.66 ± 0.07	< 0.001
BMI, kg/m^2^	24.8 ± 3.8	24.3 ± 3.7	25.2 ± 3.9	0.230
Hypertension, n (%)	71 (68.3)	29 (55.8)	42 (80.8)	0.006
Diabetes mellitus, n (%)	29 (27.9)	14 (26.9)	15 (28.8)	0.827
Smoking history, n (%)	70 (67.3)	33 (63.5)	37 (71.2)	0.403
Ischemic, n (%)	74 (71.2)	34 (65.4)	40 (76.9)	0.194
Valvular disease, n (%)	13 (12.5)	6 (11.5)	7 (13.5)	0.767
Hypertensive heart disease, n (%)	5 (4.8)	3 (5.8)	2 (3.8)	0.647
Dilated cardiomyopathy, n (%)	5 (4.8)	4 (7.7)	1 (1.9)	0.169
Hypertrophic cardiomyopathy, n (%)	6 (5.8)	3 (5.8)	3 (5.8)	1.000
Surgery, n (%)	24 (23.1)	11 (21.2)	13 (25.0)	0.642
*Resting hemodynamics*				
Heart rate, beats/min	73.0 ± 11.7	75.1 ± 10.4	71.0 ± 12.6	0.068
Systolic blood pressure, mmHg	118.8 ± 17.6	116.4 ± 16.1	121.1 ± 18.8	0.179
Diastolic blood pressure, mmHg	81.2 ± 13.3	82.0 ± 13.1	80.4 ± 13.6	0.534
*Electrocardiogram and echocardiogram*				
Sinus rhythm, n (%)	100 (96.2)	51 (98.1)	49 (94.2)	0.308
Atrial fibrillation or atrial tachycardia, n (%)	4 (3.8)	1 (1.9)	3 (5.8)	0.308
LVEF, %	53.5 ± 14.2	51.3 ± 16.2	55.8 ± 11.6	0.108
HFrEF, n (%)	17 (16.3)	11 (21.2)	6 (11.5)	0.185
HFpEF, n (%)	71 (68.3)	32 (61.5)	39 (75.0)	0.140
*Muscle strength*				
Hand grip strength, kg	34.81 ± 7.62	37.47 ± 6.32	32.15 ± 7.93	< 0.001
*Blood test*				
Hb, g/dl	13.68 ± 1.64	13.81 ± 1.69	13.55 ± 1.59	0.433
eGFR, ml/min/1.73 m^2^	60.34 ± 19.01	63.08 ± 19.75	57.60 ± 18.00	0.142
TP, g/dl	7.06 ± 0.45	7.06 ± 0.35	7.06 ± 0.54	0.967
NT-proBNP, pg/ml	839.9 [142.5, 734.8]	1042.7 [142.5, 716]	637.1 [142.5, 774]	0.772
*Medications*				
Calcium blocker, n (%)	22 (21.2)	10 (19.2)	12 (23.1)	0.631
ACE-I or ARB, n (%)	79 (76.0)	42 (80.8)	37 (71.2)	0.251
Beta-adrenergic blocking agent, n (%)	88 (84.6)	46 (88.5)	42 (80.8)	0.277
Aldosterone receptor antagonist, n (%)	24 (23.1)	11 (21.2)	13 (25.5)	0.642
ARNI, n (%)	5 (4.8)	4 (7.7)	1 (1.9)	0.169
Loop diuretic, n (%)	12 (11.5)	7 (13.5)	5 (9.6)	0.539
Tolvaptan, n (%)	2 (1.9)	1 (1.9)	1 (1.9)	1.000
SGLT2, n (%)	10 (9.6)	5 (9.6)	5 (9.6)	1.000

Data are presented as the mean ± SD for normally distributed variables and the median [25th, 75th percentile] for non-normally distributed continuous variables. ACE-I, angiotensin-converting enzyme inhibitor; ARB, angiotensin receptor blocker; ARNI, Angiotensin receptor-neprilysin inhibitor; BMI, body mass index; eGFR, estimated glomerular filtration rate; Hb, hemoglobin; HFpEF, heart failure with preserved ejection fraction; HFrEF, heart failure with reduced ejection fraction; LVEF, left ventricular ejection fraction; MWS, maximum walking speed; NT-proBNP, N-terminal pro-B-type natriuretic peptide; SD, standard deviation; SGLT2, sodium-glucose cotransporter 2 inhibitor; TP, total protein

MWS was measured by two independent physicians as observers. Intra-observer reproducibility assessed by paired-samples t-test was *p* = 0.152. Inter-observer reliability evaluated by the Pearson correlation coefficient with 20 randomly selected patients was r = 0.705 (*p* < 0.001). We could not find any reports of mean MWS in patients with heart failure. Therefore, we divided the participants based on median MWS (2.278 m/s) into the high MWS group (n = 52) and low MWS group (n = 52) to understand the clinical background associated with MWS. The high MWS group was more likely to have younger participants (54.8 ± 12.6 years vs. 65.6 ± 12.5 years, *p* < 0.001) and had fewer participants with hypertension (55.8% vs. 80.8%, *p* = 0.006), as well as higher height (1.70 ± 0.06 vs. 1.66 ± 0.07, *p* < 0.001) and hand grip strength (37.47 ± 6.32 vs. 32.15 ± 7.93 kg, *p* < 0.001) than the low MWS group. Only one patient with pacemaker implantation was in the high MWS group. Patients with an implantable cardioverter defibrillator or those who received cardiac resynchronization therapy were not present in this study.

The CPX responses are summarized in Table 2. The mean peak VO_2_ was 20.78 ± 5.03 ml/kg^/^min, the mean percent-predicted peak VO_2_ was 83.54 ± 16.49%, the mean AT was 14.39 ± 3.10 ml/kg^/^min, the mean percent-predicted AT was 92.37 ± 18.97%, and VE/VCO_2_ slope was 33.60 ± 6.05. The peak respiratory exchange ratio was 1.15 ± 0.10, while the peak Borg scale score was 16.88 ± 0.63. Median MWS-based group-wise comparisons revealed that the high MWS group demonstrated higher peak VO_2_ (22.73 ± 5.16 vs. 18.82 ± 4.09 ml/kg/min, *p* < 0.001), percent-predicted peak VO_2_ (88.40 ± 15.10% vs. 78.67 ± 16.53%, *p* = 0.002), AT (15.35 ± 3.28 vs. 13.43 ± 2.60 ml/kg/min, *p* = 0.001), and percent-predicted AT (98.52 ± 19.12% vs. 86.21 ± 16.85%, *p* < 0.001). The mean peak respiratory exchange ratio was higher in the high MWS group but exceeded 1.1 in both groups (1.18 ± 0.09 vs. 1.12 ± 0.09, *p* = 0.002). The peak Borg scale score did not differ significantly between the two groups (16.87 ± 0.74 vs. 16.90 ± 0.50, *p* = 0.756).

**Table 2:** Cardiopulmonary exercise testing responses of the study population

Characteristics	All patients (n = 104)	High MWS (n = 52)	Low MWS (n = 52)	*p* value
Peak VO_2_, ml/kg/min	20.78 ± 5.03	22.73 ± 5.16	18.82 ± 4.09	< 0.001
Percent-predicted peak VO_2_, %	83.54 ± 16.49	88.40 ± 15.10	78.67 ± 16.53	0.002
Percent-predicted peak VO_2_ ≥ 80%, n (%)	63 (60.6)	37 (71.2)	26 (50.0)	0.027
AT, ml/kg/min	14.39 ± 3.10	15.35 ± 3.28	13.43 ± 2.60	0.001
Percent-predicted AT, %	92.37 ± 18.97	98.52 ± 19.12	86.21 ± 16.85	< 0.001
Percent-predicted AT ≥ 80%, n (%)	77 (74.0)	46 (88.5)	31 (59.6)	< 0.001
VE/VCO_2_ slope	33.60 ± 6.05	32.70 ± 5.13	34.49 ± 6.78	0.133
Peak respiratory exchange ratio	1.15 ± 0.10	1.18 ± 0.09	1.12 ± 0.09	0.002
Peak Borg scale score	16.88 ± 0.63	16.87 ± 0.74	16.90 ± 0.50	0.756

Data are presented as the mean ± SD. Percent-predicted, expressed as a percentage of the baseline value defined for each sex and age group. The lower limit of normal is 80%. AT, anaerobic threshold; MWS, maximum walking speed; VO_2_, oxygen uptake; SD, standard deviation; VE/VCO_2_ slope, minute ventilation/carbon dioxide production slope

### Outcome of the relationship between MWS and CPX responses

**Fig. 2:**
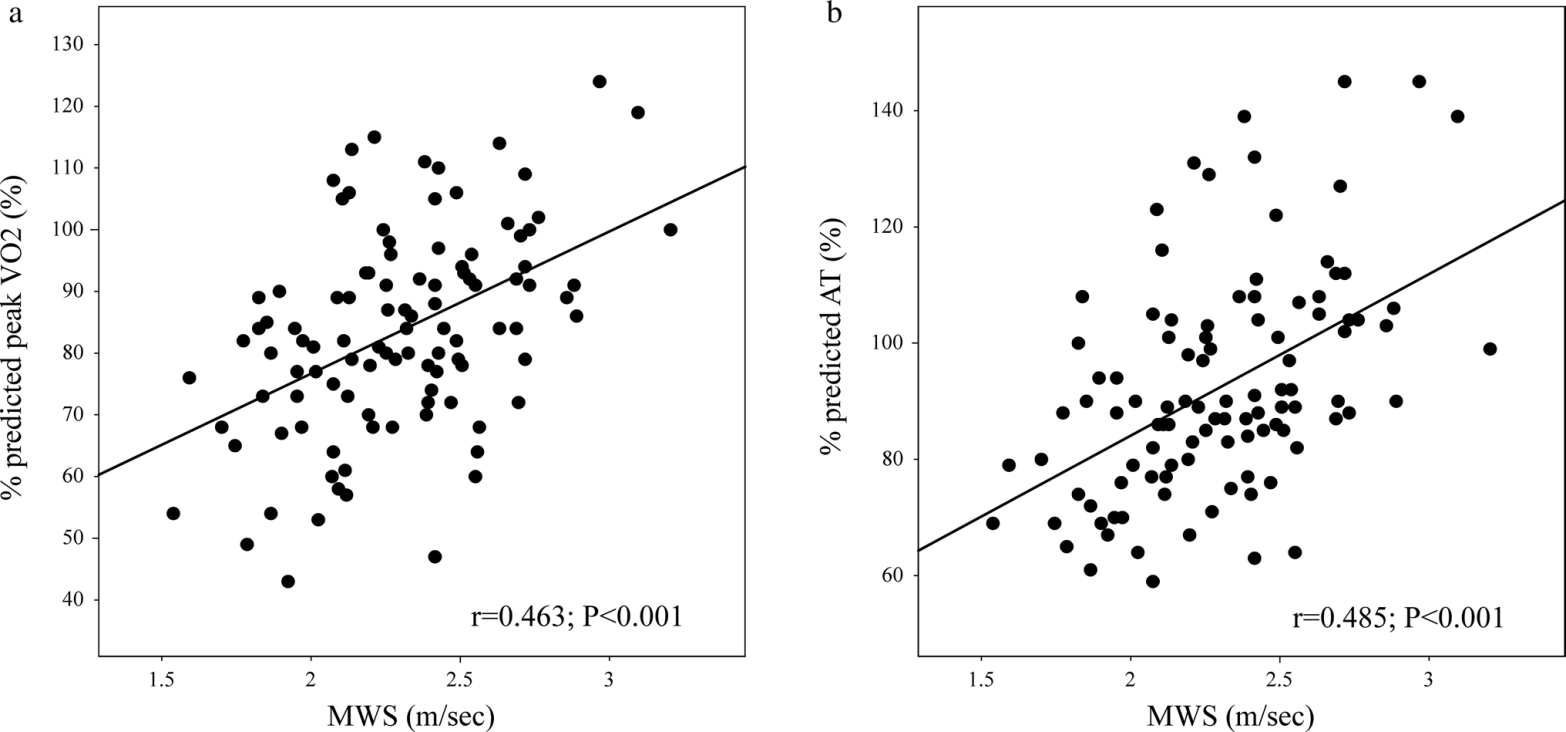
Correlation of cardiopulmonary exercise testing responses and MWS. **a** Correlation of percent-predicted peak VO_2_ and MWS. Scatterplot of MWS and percent-predicted peak VO_2_. The solid line indicates the best fit between MWS and percent-predicted peak VO_2_ (Y = 30.580 + 23.045 X. r = 0.463; *p* < 0.001). peak VO_2_, peak oxygen uptake; MWS, maximum walking speed. **b** Correlation of percent-predicted AT and MWS. Scatterplot of MWS and percent-predicted AT. The solid line indicates the best fit between MWS and percent-predicted AT (Y = 28.461 + 27.808 X. r = 0.485; *p* < 0.001). AT, anaerobic threshold; MWS, maximum walking speed

Figure 2a, b show the correlations between MWS and percent-predicted peak VO_2_ and between MWS and percent-predicted AT, respectively, in terms of the Pearson correlation coefficient. In both cases, MWS demonstrated a positive correlation (r = 0.463, *p* < 0.001; and r = 0.485, *p* < 0.001, respectively).

In simple linear regression analysis employing percent-predicted peak VO_2_ as the objective variable, MWS and hand grip strength were significantly correlated with percent-predicted peak VO_2_ (r = 0.463, *p* < 0.001; and r = 0.206, *p* = 0.036, respectively) (Table 3). In multiple linear regression analysis employing these two items as explanatory variables, only MWS demonstrated a significant positive correlation (standardized β: 0.471, *p* < 0.001) (Table 3).

**Table 3:** Simple and multiple linear regression analyses with percent-predicted peak VO_2_ as the objective variable

Variable	r	*p* value
*Simple linear regression analysis*		
MWS	0.463	< 0.001
Age	− 0.098	0.321
Hand grip strength	0.206	0.036
Hypertension	− 0.174	0.077
Height	0.101	0.306
Smoking history	− 0.120	0.227
Resting heart rate	− 0.059	0.555
Ischemic	0.122	0.218
LVEF	0.187	0.058
Hb	0.065	0.513
eGFR	0.178	0.070
TP	− 0.091	0.377
NT-proBNP	− 0.032	0.744

Variable	Standardized β	*p* value
*Multiple linear regression analysis*		
MWS	0.471	< 0.001
Hand grip strength	− 0.018	0.859

Percent-predicted, expressed as a percentage of the baseline value defined for each sex and age group. The lower limit of normal is 80%. eGFR, estimated glomerular filtration rate; Hb, hemoglobin; LVEF, left ventricular ejection fraction; MWS, maximum walking speed; NT-proBNP, N-terminal pro-B-type natriuretic peptide; TP, total protein; VO_2_, oxygen uptake

In simple linear regression analysis employing percent-predicted AT as the objective variable, MWS, age, hand grip strength, hypertension, and smoking history demonstrated significant correlations (r = 0.485, *p* < 0.001; r = − 0.353, *p* < 0.001; r = 0.350, *p* < 0.001; r = − 0.251, *p* = 0.010; and r = − 0.199, *p* = 0.042, respectively) (Table 4). In multiple linear regression analysis employing these items as explanatory variables, only MWS demonstrated a significant positive correlation (standardized β: 0.362, *p* < 0.001) (Table 4).

**Table 4:** Simple and multiple linear regression analyses with percent-predicted AT as the objective variable

Variable	r	*p* value
*Simple linear regression analysis*		
MWS	0.485	< 0.001
Age	− 0.353	< 0.001
Hand grip strength	0.350	< 0.001
Hypertension	− 0.251	0.010
Height	0.173	0.080
Smoking history	− 0.199	0.042
Resting heart rate	0.077	0.435
Ischemic	0.034	0.734
LVEF	0.009	0.928
Hb	0.142	0.150
eGFR	0.151	0.127
TP	0.051	0.619
NT-proBNP	0.061	0.540

Variable	Standardized β	*p* value
Multiple linear regression analysis		
MWS	0.362	< 0.001
Age	− 0.076	0.493
Hand grip strength	0.106	0.322
Hypertension	− 0.080	0.391
Smoking history	− 0.127	0.147

Percent-predicted, expressed as a percentage of the baseline value defined for each sex and age group. The lower limit of normal is 80%. AT, anaerobic threshold; eGFR, estimated glomerular filtration rate; Hb, hemoglobin; LVEF, left ventricular ejection fraction; MWS, maximum walking speed; NT-proBNP, N-terminal pro-B-type natriuretic peptide; TP, total protein

In logistic regression analyses employing an 80% cutoff in percent-predicted peak VO_2_, only MWS demonstrated a significant association (odds ratio [OR]: 1.258, 95% confidence interval [CI]: 1.092–1.448, *p* = 0.001), while hypertension demonstrated trends toward significant associations (OR: 0.457, 95% CI: 0.186–1.121, *p* = 0.087) (Table 5). In multiple logistic regression analyses employing these items as explanatory variables, only MWS was identified as a significant factor (OR: 1.239, 95% CI: 1.071–1.432, *p* = 0.004) (Table 5).

**Table 5:** Logistic regression analyses with an 80% cutoff in percent-predicted peak VO_2_

Variable	OR	95% CI	*p* value
*Univariate analysis*			
MWS (0.1 m/s)	1.258	1.092–1.448	0.001
Age	0.989	0.961–1.019	0.475
Hand grip strength	1.028	0.975–1.085	0.305
Hypertension	0.457	0.186–1.121	0.087
Height (0.1 m)	0.929	0.528–1.633	0.797
Smoking history	0.771	0.330–1.803	0.549
Resting heart rate	0.987	0.954–1.021	0.438
Ischemic	1.256	0.531–2.973	0.604
LVEF	1.020	0.992–1.049	0.162
Hb	1.005	0.790–1.279	0.967
eGFR	1.007	0.986–1.028	0.527
TP	1.144	0.464–2.817	0.770
NT-proBNP	1.000	1.000–1.000	0.538
*Multivariable analysis*			
MWS (0.1 m/s)	1.239	1.071–1.432	0.004
Hypertension	0.662	0.253–1.731	0.401

Percent-predicted, expressed as a percentage of the baseline value defined for each sex and age group. The lower limit of normal is 80%. CI, confidence interval; eGFR, estimated glomerular filtration rate; Hb, hemoglobin; LVEF, left ventricular ejection fraction; MWS, maximum walking speed; NT-proBNP, N-terminal pro-B-type natriuretic peptide; OR, odds ratio; TP, total protein; VO_2_, oxygen uptake

In logistic regression analyses employing an 80% cutoff in percent-predicted AT, only MWS and hand grip strength demonstrated a significant association (OR: 1.478, 95% CI: 1.221–1.788, *p* < 0.001 and OR: 1.081, 95% CI: 1.011–1.157, *p* = 0.023, respectively) (Table 6). In the multiple logistic regression analysis employing these items as explanatory variables, only MWS was identified as a significant factor (OR: 1.469, 95% CI: 1.194–1.807, *p* < 0.001) (Table 6).

**Table 6:** Logistic regression analyses with an 80% cutoff in percent-predicted AT

Variable	OR	95% CI	*p* value
*Univariate analysis*			
MWS (0.1 m/s)	1.478	1.221–1.788	< 0.001
Age	0.975	0.942–1.008	0.140
Hand grip strength	1.081	1.011–1.157	0.023
Hypertension	0.529	0.191–1.469	0.222
Height (0.1 m)	1.068	0.570–1.999	0.838
Smoking history	0.826	0.319–2.139	0.694
Resting heart rate	1.002	0.965–1.041	0.919
Ischemic	0.822	0.306–2.210	0.697
LVEF	1.005	0.975–1.036	0.749
Hb	1.082	0.828–1.415	0.563
eGFR	1.009	0.986–1.033	0.422
TP	1.036	0.378–2.838	0.946
NT-proBNP	1.000	1.000–1.000	0.835
*Multivariable analysis*			
MWS (0.1 m/s)	1.469	1.194–1.807	< 0.001
Hand grip strength	1.005	0.936–1.079	0.883

Percent-predicted, expressed as a percentage of the baseline value defined for each sex and age group. The lower limit of normal is 80%. AT, anaerobic threshold; CI, confidence interval; eGFR, estimated glomerular filtration rate; Hb, hemoglobin; LVEF, left ventricular ejection fraction; MWS, maximum walking speed; NT-proBNP, N-terminal pro-B-type natriuretic peptide; OR, odds ratio; TP, total protein

### Outcomes via PSM

In addition, PSM was examined between the high and low MWS groups to minimize the influence of potentially confounding variables that could lead to biased results (Fig. 1). The variables used to generate propensity scores were age, height, hypertension, heart failure with reduced ejection fraction, and heart failure with preserved ejection fraction based on the results of the pre-matching two-group comparison. Online Resource 1 shows the baseline characteristics after PSM. Finally, 31 matched pairs were generated, and baseline characteristics were well-balanced between the two groups.

Median MWS-based group-wise comparisons after PSM indicated that the high MWS group demonstrated higher peak VO_2_ (22.09 ± 5.28 vs. 19.54 ± 4.21 ml/kg/min, *p* = 0.040) and percent-predicted peak VO_2_ (89.65 ± 14.67% vs. 78.35 ± 16.59%, *p* = 0.006) (Online Resource 2). Similarly, AT and percent-predicted AT kept trending higher in the high MWS group (15.03 ± 2.98 vs. 13.30 ± 2.76 ml/kg/min, *p* = 0.060 and 96.29 ± 16.22% vs. 86.26 ± 17.97%, *p* = 0.070, respectively), and the percentage of percent-predicted AT ≥ 80% was significantly higher in the high MWS group (93.5% vs. 64.5%, *p* = 0.005) (Online Resource 2).

Online Resource 3-a and 3-b depict the Pearson correlation coefficient-based correlations after PSM between MWS and percent-predicted peak VO_2_ and between MWS and percent-predicted AT, respectively. In both instances, a positive correlation was observed for MWS (r = 0.484, *p* < 0.001; and r = 0.434, *p* < 0.001, respectively).

In logistic regression analyses conducted after PSM using an 80% cutoff in percent-predicted peak VO_2_ and AT, only MWS showed a significant association. The ORs for MWS were 1.311 (95% CI: 1.069–1.607, *p* = 0.009) for percent-predicted peak VO_2_ and 1.559 (95% CI: 1.153–2.107, *p* = 0.004) for percent-predicted AT (Online Resource 4-a and 4-b).

## Discussion

In the present study, we assessed the relationships of MWS with peak VO_2_ and AT by CPX in male patients with heart failure. We found that MWS demonstrated significant positive correlations with percent-predicted peak VO_2_ and percent-predicted AT. Medications, medical history, underlying heart disease, left ventricular ejection fraction, height, or blood test findings, such as N-terminal pro-B-type natriuretic peptide, did not affect these correlations. Our results also indicated that the representative CPX indicators percent-predicted peak VO_2_ and percent-predicted AT can be predicted with MWS. Additionally, similar results were also obtained after PSM. To our knowledge, this is the first-ever study to demonstrate that a 5-m walking speed is associated with peak VO_2_ and AT in patients with heart failure.

### Mechanisms

Exercise capacity predicts survival in patients with cardiovascular disease, including heart failure [25], while gait speed has been shown to have a prognostic capability [9, 10]. These findings mean that walking is the foundation of exercise. The preventive effect of walking regarding lifestyle diseases and coronary artery disease positively correlates with exercise quantity, i.e., the amount of physical activity (intensity × duration) [26]. Walking consists of two factors: speed and distance. Moreover, a previous study reported that brisk walkers had longer average life expectancies [27]. The results of the present study, which found MWS to be correlated with peak VO_2_ and AT, are consistent with those reported by the abovementioned studies.

### Advantages of MWS in clinical practice

Although CPX is practically the only test that accurately measures peak VO_2_ and AT, it can only be performed at a limited number of centers since it requires staff with specialized skills and knowledge and specialized equipment and takes 1 h to perform. Consequently, simpler alternatives for estimating peak VO_2_ and AT are sought, such as the 6MWT [11, 12] and the shuttle walking test [13, 14]. However, in addition to being cumbersome, taking a long time to perform, and requiring a facility with a roughly 30-m straight line, the 6MWT does not quantify loads, resulting in problems with reproducibility and standardization. The 6MWT is also limited in objectivity and accuracy as results vary due to factors such as encouragement, mood, and familiarity with the test [12]. The shuttle walking test quantifies loads unlike the 6MWT, and as for VO_2_ dynamics, it can assess cardiopulmonary function in the same manner as the 6MWT without major differences [28]. However, the test requires up to 20 min to complete, and there is currently little evidence regarding its applicability for assessing exercise tolerance in patients with heart disease. Additionally, these field walking tests cannot be performed with hearing-impaired patients, and they do not allow for monitoring a 12-lead ECG or blood pressure over time during walking. In contrast, the 5-m walk test, as was used in the present study, enables the measurement of gait speed in everyday clinical settings without the need for special knowledge or equipment and can be conducted in a short time and a small space. Additionally, the 5-m walk test is easy to explain and can be safely performed repeatedly, making it reproducible and highly objective. Although the measurement of gait speed over a short distance cannot be compared easily with a long-distance walking test down a corridor, our results suggested that the ability to estimate peak VO_2_ and AT with MWS makes the 5-m walk test effective as a simple test for estimating exercise tolerance. Further, although the 6MWT and the shuttle walking test are representative tolerance tests and remain important, MWS can be conducted easily at any facility in a short time without the need for specialized knowledge, meaning that measurement of MWS over 5 m may be important as an initial screening test. Furthermore, the patients experienced little anxiety, did not refuse the test thanks to its short duration, and did not demonstrate any adverse events. These points may also make the 5-m walk test effective as an initial screening test. Additionally, in light of a previous study that reported that grading based on peak VO_2_, AT, and VE/VCO_2_ slope enables stratification of the risks of mortality and heart failure [3, 29, 30], MWS also has promise as a useful screening test for risk assessment.

### Application to cardiac rehabilitation

Cardiac rehabilitation is a well-established non-pharmacological treatment for many patients with cardiac disease. Especially for patients with ischemic heart disease and heart failure, cardiac rehabilitation is considered a Class I therapy by the American Heart Association and European Society of Cardiology guidelines [31–35].

The recommended exercise for cardiac rehabilitation is aerobic exercise, such as walking and cycling. Aerobic exercise does not cause intramuscular lactate accumulation and does not lead to acidosis [36]; it also preserves the contractile response of the heart and does not cause a marked increase in blood catecholamines [36, 37]. In other words, aerobic exercise is recommended because it is safe and can be performed for extended periods of time.

Therefore, the estimation of AT, the limiting value of aerobic exercise, is an important indicator in safe and effective cardiac rehabilitation. Contrarily, it is practically difficult to perform CPX on all patients, and AT cannot be measured in medical facilities that do not have CPX.

However, the results of this study indicate the possibility of using MWS as a screening test to estimate AT and peak VO_2_. MWS is a simple, safe, inexpensive, and repeatable test that can be performed by non-physician staff. Therefore, screening for exercise tolerance and risk in cardiac rehabilitation, similar to CPX, can be possible in facilities that do not have specialized equipment. Furthermore, the results are expected to contribute to greater social recognition and dissemination of cardiac rehabilitation, similar to frailty and sarcopenia.

### Limitations

There are limitations to consider in this study. First, this is a single-center, retrospective observational study with small sample size. The clinical background of this study differs from that of Japanese registry studies of heart failure, such as ATTEND Registry and JCARE-CARD [38, 39]. Therefore, the possibility of selection bias cannot be ruled out. However, to minimize the influence of bias, we performed PSM and reevaluated the relationship between MWS and % predicted peak VO_2_ and AT after PSM. The results also showed that MWS had a significant positive correlation with predicted peak VO_2_ and predicted AT as well as before PSM.

Second, it is unclear whether endurance and response to long-term loading were properly assessed in the measurement of gait speed over the course of a few seconds. In particular, it is unclear whether the risk of myocardial ischemia was sufficiently assessed; thus, we inspected participants’ medical records and conducted diagnostic interviews regarding their conditions before testing. In the 5-m walk test and CPX, none of the participants presented with findings indicative of ischemia. However, despite these limitations, this is the first significant report showing a positive correlation of MWS with peak VO_2_ and AT in patients with heart failure. Moreover, the ability to estimate peak VO_2_ and AT in a simple fashion with MWS as a screening test was suggested to be extremely effective in providing safe cardiac rehabilitation.

### Conclusion

We found MWS to be strongly correlated with peak VO_2_ and AT in male patients with heart failure. Measurement of MWS as a screening test for exercise tolerance may provide a simple means of estimating peak VO_2_ and AT in heart failure patients. A prospective multicenter study needs to be conducted based on the results of the present study.

### Supplementary Information

Below is the link to the electronic supplementary material.

Supplementary file1 (PDF 2482 KB)

## Data Availability

The datasets generated and analyzed during the current study are available from the corresponding author on reasonable request.
