# Supplementary material for: Relationship of maximum walking speed with peak oxygen uptake and anaerobic threshold in male patients with heart failure
Source: Heart Vessels. 2023 Jul 26;38(11):1344–55. doi: 10.1007/s00380-023-02289-y (PMC10520159; doi:10.1007/s00380-023-02289-y)
Supplement: Supplementary file 1 — Supplementary file1 (PDF 2482 KB) [file 380_2023_2289_MOESM1_ESM.pdf]

Relationship of maximum walking speed with peak oxygen uptake and anaerobic threshold in male patients with heart failure

Heart and Vessels

Masahiro Koen, Yoshiaki Kubota, Miwa Tokita, Kazuyo Kato, Hiroshi Takahashi, Koichi Akutsu, Kuniya Asai, Hitoshi Takano

Department of Cardiovascular Medicine, Nippon Medical School, Tokyo, Japan

Corresponding Author

E-mail: htakano@nms.ac.jp

Online Resource 1. Clinical characteristics of the study population after PSM

Characteristics	All patients (n = 62)	High MWS (n = 31)	Low MWS (n = 31)	p-value
Age, years	59.8 ± 11.1	60.2 ± 11.3	59.4 ± 11.0	0.768
Height, m	1.69 ± 0.06	1.69 ± 0.05	1.69 ± 0.06	0.993
BMI, kg/m^2	25.7 ± 4.0	25.0 ± 3.3	26.4 ± 4.4	0.163
Hypertension, n (%)	46 (74.2)	23 (74.2)	23 (74.2)	1.000
Diabetes mellitus, n (%)	20 (32.3)	10 (32.3)	10 (32.3)	1.000
Smoking history, n (%)	45 (72.6)	21 (67.7)	24 (77.4)	0.393
Ischemic, n (%)	44 (71.0)	21 (67.7)	23 (74.2)	0.576
Valvular disease, n (%)	5 (8.1)	2 (6.5)	3 (9.7)	0.641
Hypertensive heart disease, n (%)	5 (8.1)	3 (9.7)	2 (6.5)	0.641
Dilated cardiomyopathy, n (%)	2 (3.2)	1 (3.2)	1 (3.2)	1.000
Hypertrophic cardiomyopathy, n (%)	4 (6.5)	3 (9.8)	1 (3.2)	0.301
Surgery, n (%)	15 (24.2)	6 (19.4)	9 (29.0)	0.374
Resting hemodynamics				
Heart rate, beats/min	73.7 ± 11.3	74.5 ± 10.4	72.8 ± 12.2	0.541
Systolic blood pressure, mmHg	117.1 ± 16.5	116.9 ± 14.8	117.4 ± 18.3	0.921

Diastolic blood pressure, mmHg	80.9 ± 13.3	81.4 ± 12.0	80.4 ± 14.8	0.764
Electrocardiogram and echocardiogram				
Sinus rhythm, n (%)	60 (96.8)	30 (96.8)	30 (96.8)	1.000
Atrial fibrillation or atrial tachycardia, n (%)	2 (3.2)	1 (3.2)	1 (3.2)	1.000
LVEF, %	54.8 ± 13.2	53.8 ± 14.2	55.8 ± 12.3	0.555
HFrEF, n (%)	10 (16.1)	6 (19.3)	4 (12.9)	0.490
HfpEF, n (%)	42 (67.7)	20 (64.5)	22 (71.0)	0.587
Muscle strength				
Hand grip strength, kg	36.16 ± 7.29	37.53 ± 5.72	34.79 ± 8.44	0.140
Blood test				
Hb, g/dl	13.66 ± 1.46	13.84 ± 1.53	13.49 ± 1.38	0.355
eGFR, ml/min/1.73 m^2	60.15 ± 17.21	60.23 ± 17.31	60.06 ± 17.40	0.971
TP, g/dl	7.04 ± 0.46	7.03 ± 0.41	7.04 ± 0.51	0.879
NT-proBNP, pg/ml	873.1 [136.3, 699.3]	1138.3 [181.5, 642.5]	608.0 [121.5, 771.0]	0.394
Medications				
Calcium blocker, n (%)	14 (22.6)	7 (22.6)	7 (22.6)	1.000
ACE-I or ARB, n (%)	50 (80.6)	26 (83.9)	24 (77.4)	0.520

Beta-adrenergic blocking agent, n (%)	52 (83.9)	27 (87.1)	25 (80.6)	0.490
Aldosterone receptor antagonist, n (%)	13 (21.0)	4 (12.9)	9 (29.0)	0.119
ARNI, n (%)	3 (4.8)	2 (6.5)	1 (3.2)	0.554
Loop diuretic, n (%)	7 (11.3)	3 (9.7)	4 (12.9)	0.688
Tolvaptan, n (%)	2 (3.2)	1 (3.2)	1 (3.2)	1.000
SGLT2, n (%)	7 (11.3)	2 (6.5)	5 (16.1)	0.229

Data are presented as the mean ± SD for normally distributed variables and the median [25th, 75th percentile] for non-normally distributed continuous variables. ACE-I, angiotensin-converting enzyme inhibitor; ARB, angiotensin receptor blocker; ARNI, Angiotensin receptor-neprilysin inhibitor; BMI, body mass index; eGFR, estimated glomerular filtration rate; Hb, hemoglobin; HfpEF, heart failure with preserved ejection fraction; HfrEF, heart failure with reduced ejection fraction; LVEF, left ventricular ejection fraction; MWS, maximum walking speed; NT-proBNP, N-terminal pro-B-type natriuretic peptide; PSM, propensity score matching; SD, standard deviation; SGLT2, sodium-glucose cotransporter 2 inhibitor; TP, total protein.

Online Resource 2. Cardiopulmonary exercise testing responses of the study population after PSM

Characteristics	All patients (n = 62)	High MWS (n = 31)	Low MWS (n = 31)	p-value
Peak VO_2, ml/kg/min	20.81 ± 4.91	22.09 ± 5.28	19.54 ± 4.21	0.040
Percent-predicted peak VO_2, %	84.00± 16.54	89.65 ± 14.67	78.35 ± 16.59	0.006
Percent-predicted peak VO_2 ≥ 80%, n (%)	40 (64.5)	24 (77.4)	16 (51.6)	0.034
AT, ml/kg/min	14.33 ± 2.94	15.03 ± 2.98	13.30 ± 2.76	0.060
Percent-predicted AT, %	92.27 ± 17.45	96.29 ± 16.22	88.26 ± 17.97	0.070
Percent-predicted AT ≥ 80%, n (%)	49 (79.0)	29 (93.5)	20 (64.5)	0.005
VE/VCO_2 slope	33.10 ± 5.09	33.70 ± 5.30	32.49 ± 4.87	0.351
Peak respiratory exchange ratio	1.14 ± 0.09	1.18 ± 0.08	1.11 ± 0.09	0.005
Peak Borg scale score	16.89 ± 0.52	16.94 ± 0.36	16.84 ± 0.64	0.464

Data are presented as the mean ± SD. Percent-predicted, expressed as a percentage of the baseline value defined for each sex and age group. The lower limit of normal is 80%. AT, anaerobic threshold; MWS, maximum walking speed; VO_2, oxygen uptake; PSM, propensity score matching; SD, standard deviation; VE/VCO_2 slope, minute ventilation/carbon dioxide production slope.

Online Resource 3. Correlation of cardiopulmonary exercise testing responses and MWS after PSM

a. Correlation of percent-predicted peak VO_2 and MWS after PSM

Scatterplot of MWS and percent-predicted peak VO_2 after propensity score matching. The solid line indicates the best fit between MWS and percent-predicted peak VO_2 ($Y = 27.845 + 24.091 X$. $r = 0.484$; $p < 0.001$).

Peak VO_2, peak oxygen uptake; MWS, maximum walking speed; PSM, propensity score matching.

b. Correlation of percent-predicted AT and MWS after PSM. Scatterplot of MWS and percent-predicted AT after propensity score matching. The solid line indicates the best fit between MWS and percent-predicted AT ($Y = 39.128 + 22.800 X$. $r = 0.434$; $p < 0.001$).

AT, anaerobic threshold; MWS, maximum walking speed; PSM, propensity score matching.

Online Resource 3-a

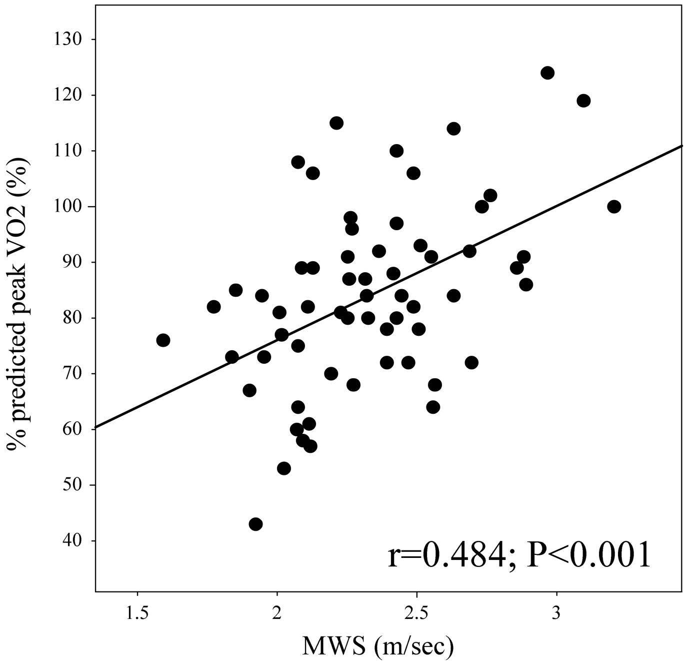

Online Resource 3-b

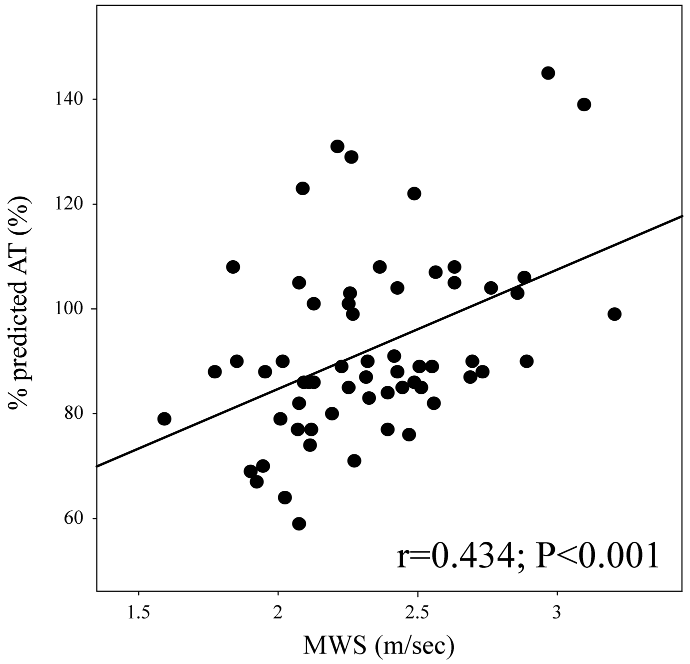

Online Resource 4. Logistic regression analyses with an 80% cutoff in percent-predicted peak VO_2 and AT after PSM

a. Percent-predicted peak VO_2

**Univariate analysis**			
Variable	OR	95% CI	p-value
MWS (0.1 m/s)	1.311	1.069−1.607	0.009
Hand grip strength	1.002	0.932−1.077	0.962
Smoking history	0.462	0.130−1.643	0.233
Resting heart rate	0.983	0.938−1.030	0.473
Ischemic	0.875	0.275−2.782	0.821
LVEF	1.025	0.985−1.067	0.231
Hb	0.950	0.661−1.365	0.781
eGFR	0.997	0.966−1.028	0.830
TP	1.167	0.353−3.865	0.800
NT-proBNP	1.000	1.000−1.000	0.866

Percent-predicted, expressed as a percentage of the baseline value defined for each sex and age group. The lower limit of normal is 80%. CI, confidence interval; eGFR, estimated glomerular filtration rate; Hb, hemoglobin; LVEF, left ventricular ejection

fraction; MWS, maximum walking speed; NT-proBNP, N-terminal pro-B-type natriuretic peptide; OR, odds ratio; PSM, propensity score matching; TP, total protein; VO_2, oxygen uptake.

b. Percent-predicted AT

Univariate analysis			
Variable	OR	95% CI	p-value
MWS (0.1 m/s)	1.559	1.153−2.107	0.004
Hand grip strength	1.021	0.936−1.115	0.635
Smoking history	0.412	0.081−2.092	0.285
Resting heart rate	0.998	0.945−1.054	0.947
Ischemic	1.111	0.294−4.205	0.877
LVEF	0.986	0.939−1.035	0.560
Hb	1.077	0.709−1.636	0.727
eGFR	0.978	0.939−1.019	0.285
TP	1.015	0.250−4.114	0.984
NT-proBNP	1.000	1.000−1.000	0.691

Percent-predicted, expressed as a percentage of the baseline value defined for each sex and age group. The lower limit of normal is 80%. AT, anaerobic threshold; CI, confidence interval; eGFR, estimated glomerular filtration rate; Hb, hemoglobin; LVEF, left ventricular ejection fraction; MWS, maximum walking speed; NT-proBNP, N-terminal pro-B-type natriuretic peptide; OR, odds ratio; PSM, propensity score

matching; TP, total protein.
